# Agility testing in amateur soccer: A pilot study of selected physical and perceptual-cognitive contributions

**DOI:** 10.1371/journal.pone.0253819

**Published:** 2021-06-24

**Authors:** Stefan Altmann, Rainer Neumann, Sascha Härtel, Gunther Kurz, Thorsten Stein, Alexander Woll

**Affiliations:** 1 Department for Performance Analysis, Institute of Sports and Sports Science, Karlsruhe Institute of Technology, Karlsruhe, Germany; 2 TSG ResearchLab gGmbH, Zuzenhausen, Germany; 3 Institute of Movement and Sport, University of Education Karlsruhe, Karlsruhe, Germany; 4 TSG 1899 Hoffenheim, Zuzenhausen, Germany; 5 BioMotion Center, Institute of Sports and Sports Science, Karlsruhe Institute of Technology, Karlsruhe, Germany; 6 Department for Social and Health Sciences in Sport, Institute of Sports and Sports Science, Karlsruhe Institute of Technology, Karlsruhe, Germany; Universidade Federal de Mato Grosso do Sul, BRAZIL

## Abstract

The purpose of this study was to examine the relationships of physical and perceptual-cognitive factors with agility performance in amateur soccer players. Fifteen male amateur soccer players (age, 24.5 ± 1.9 years) completed a linear-sprint test with splits at 5 m, 10 m, and 30 m, a change-of-direction test of 12 m with 2 pre-planned directional changes of 45° at 2 m and 7 m, and a soccer-specific agility test with same movement pattern as the change-of-direction test but with the inclusion of a human stimulus performing passing movements. Additionally, the perceptual-cognitive deficit (agility performance minus change-of-direction performance) was calculated. In relation to agility performance, linear-sprint performance showed large relationships, which were higher with increasing sprint distance (5 m, r = 0.57; 10 m, r = 0.59; 30 m, r = 0.69), change-of-direction performance a very large relationship (r = 0.77), and the perceptual-cognitive deficit a large relationship (r = 0.55). The findings of this study highlight the relatively high contribution of both physical (i.e., linear-sprint and change-of-direction performance) and perceptual-cognitive factors (i.e., perceptual-cognitive deficit) in relation to soccer-specific agility performance at an amateur level. Consequently, such elements might be recommended to be included in training programs aimed at improving agility performance at this playing level. Moreover, the here introduced perceptual-cognitive deficit allows for a convenient and likewise thorough analysis of agility performance. Future studies should investigate the effects of both physically and perceptual-cognitive oriented training interventions on agility performance, which is considered a key element for success in soccer.

## Introduction

Speed in its different facets is considered a crucial component of overall performance in team-sports such as soccer [1, 2]. Throughout a soccer match, players perform numerous accelerations and sprints at maximal speed [3], both with and without changes of direction [4, 5]. Such actions seldom occur in a pre-planned manner but rather in response to a stimulus, thereby taxing not only the physical but also the perceptual-cognitive skills (e.g., anticipation and decision making) of the players [6]. In this regard, changing speed or direction in response to a stimulus has been termed “agility” [7]. Game situations where agility is vital are manifold, including intercepting or reaching a pass, evading an opponent, and creating or closing space between oneself and an opponent [8].

Following its importance in soccer, a number of agility tests has been introduced into research and practice in recent years [6, 9, 10]. Besides a rather general agility test for invasion sports [11, 12], tests aiming to more closely reflect the demands placed upon soccer players are gaining in popularity [13–17]. In order to obtain a better understanding of the factors that underpin agility performance in these tests, researchers have predominantly investigated its relationships with physical skills such as linear-sprint performance over various distances and change-of-direction performance. More specifically, Krolo et al. [17] reported moderate correlations (r = 0.36–0.49) for 10-m and 20-m linear-sprint times with agility performance in high-level youth soccer players. In addition, large to very large correlations (r = 0.50–0.85) have been reported regarding change-of-direction time, again in samples of high-level youth soccer players [14, 16, 17]. In contrast, only small relationships between change-of-direction time and agility performance were evident in a study of amateur adult soccer players [15].

While providing first insights into factors contributing to agility performance, a drawback of these studies is that the applied agility tests use numbers on a screen or flashing lights as a stimulus to which the players are required to respond to. In particular, research has shown that such non-specific stimuli do not allow players to deploy their anticipation skills but rather force them to simply react [18]. Practically speaking, while higher-level athletes are able to make use of anticipatory kinematic cues during a sport-specific scenario, a light is simply either on or off, thereby only assessing the athletes’ speed of information processing [6]. As a result, perceptual-cognitive factors that affect agility performance might have not been captured in the abovementioned studies due to the non-specific stimuli applied in the respective agility tests [10]. Therefore, tests using such stimuli might be not optimal for investigating factors that underpin agility in soccer-specific scenarios.

To overcome this issue, an agility test tailored to the requirements of soccer has recently been developed [19]. In this test, the stimulus is provided by a live tester, who performs soccer-specific passing movements. To date, little is known about the factors that contribute to agility performance in this specific test.

Consequently, the purpose of this study was to examine the relationships of physical and perceptual-cognitive factors with agility performance in the newly developed soccer-specific agility test. Based on previous research, we hypothesized that both physical and perceptual-cognitive factors are correlated with agility performance. The results of this study could broaden our understanding of agility in soccer and support researchers and coaches in designing training programs aiming to improve agility performance.

## Materials and methods

### Study design

In the present cross-sectional study, amateur soccer players completed a linear-sprint test (splits at 5 m, 10 m, and 30 m), a change-of-direction test, and a soccer-specific agility test. As an additional measure, the perceptual-cognitive deficit was calculated as the difference between the time required to complete the agility test and the change-of-direction test, respectively. All tests were conducted at the beginning of the competitive season on an artificial-grass soccer pitch.

### Subjects

Fifteen male amateur outfield soccer players with at least five years of experience competing on a regional level (age, 24.5 ± 1.9 years; age range, 21–27 years; height, 176.5 ± 7.0 cm; mass, 73.5 ± 8.8 kg; body mass index, 23.5 ± 1.9; two training sessions and one official match per week) volunteered to participate in this study. All players were free from injuries at the time of testing. The study was approved by the institutional review board of the Institute of Sports and Sports Science, Karlsruhe, Germany. All athletes gave their written informed consent before participation.

### Procedures

All players performed a standardized 15-minute warm-up including jogging, short accelerations, and movement preparation exercises. Subsequently, participants completed the three abovementioned tests in the following order: linear-sprint test (splits at 5 m, 10, and 30 m), change-of-direction test, and agility test. A 3-minute passive rest between the trials of each test and a 10-minute passive rest between the tests was provided to ensure full recovery [9].

For all tests, a wireless single-beam timing light system (TAG Heuer, La-Chaux-de-Fonds, Switzerland) was used. The initial timing lights were mounted at 0.25 m, matching approximately the ankle of the back foot’s height of the starting stance. All following timing lights were set at 1.00 m, matching approximately hip height. The starting distance from the initial timing lights was set at 0.30 m [20].

#### Linear-sprint test

**Fig 1:**
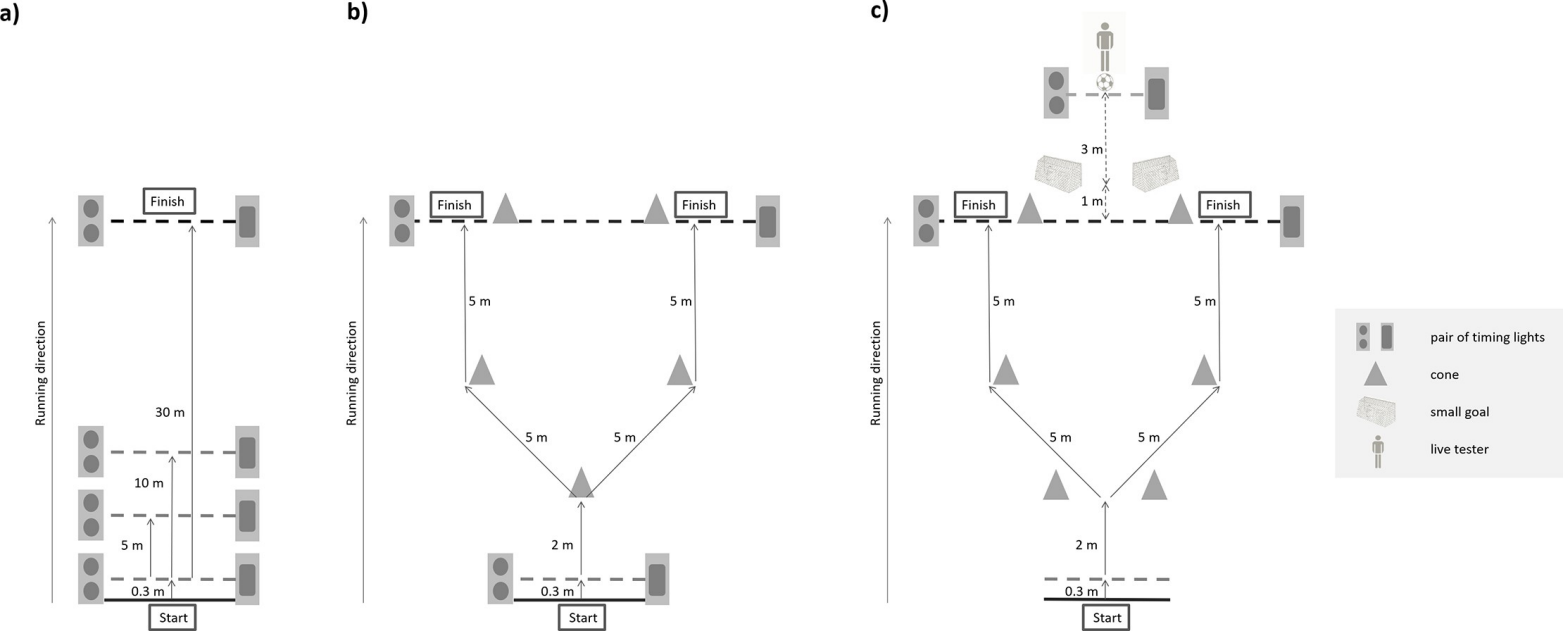
Schematic illustration of the three tests. a) Linear-sprint test (splits at 5 m, 10 m, and 30 m); b) Change-of-direction test (12 m with 2 pre-planned directional changes of 45° at 2 m and 7 m); c) Soccer-specific agility test (same movement pattern as the change-of-direction test but with the inclusion of a human stimulus performing passing movements to which the players were instructed to respond to).

Athletes performed 3 sprint trials over a distance of 30 m with splits being taken at 5 m and 10 m (Fig 1). The recorded score for this test was the mean value of the 3 trials. The reliability (intraclass correlation coefficient, ICC; coefficient of variation, CV) of the 5-m, 10-m, and 30-m sprint time in the present study was 0.83, 0.88, 0.96, and 2.10%, 1.24%, 0.67%, respectively.

#### Change-of-direction test

The change-of-direction test comprised a total distance of 12 m with 2 changes of direction of 45° at 2 m and 7 m (Fig 1). The dimensions of the change-of-direction test were exactly the same as those in the agility test (see below). Each player performed 4 trials (2 to the left, 2 to the right). Trials to the left and to the right were carried out in a counterbalanced randomized order. The mean of all 4 trials was recorded for analysis. The reliability (ICC and CV) of the change-of-direction time in the present study was 0.58–0.61 and 1.24–1.27%, respectively.

#### Agility test

The agility test used in this study has been described elsewhere in detail [19]. In brief, the players stood on the start line, with a live tester (representing an opponent) facing them at a distance of approximately 15 m. The tester had a ball under the sole of one foot. The timing began at the moment the tester touched the ball to roll forward in order to initiate one of four possible passing movements which ended by the tester passing the ball into a small goal to his left or right. The four possible passing movements were:

1. Touch the ball with the right foot, pass into the left goal with the inside of the right foot.
2. Touch the ball with the right foot, pass into the right goal with the outside of the right foot.
3. Touch the ball with the left foot, pass into the right goal with the inside of the left foot.
4. Touch the ball with the left foot, pass into the left goal with the outside of the left foot.

The athletes were instructed to move forward as soon as they recognized the ball moving (i.e., when the tester touched the ball to move forwards). Further, they should respond to the movements of the tester by changing direction into the passing direction. In doing so, they were instructed recognize and anticipate the passing direction as soon as possible, while emphasizing both decision accuracy and movement speed. After changing direction in response to the tester’s pass at an angle of 45° at approximately 2 m after the start line, the participants ran straight for 5 m. Consequently, they completed another change of direction of 45° to the other side, to finally run straight for another 5 m through the finish timing lights (Fig 1). Hence, the timing began by the rolling ball triggering the timing lights in front of the tester and ended by the player running through the finish timing lights. Each participant completed all four passing movements once in a balanced randomized order. The athletes did not know how many passing movements existed, nor how they would be executed.

The four possible passing movements place unique demands on the players and were repeated for only one time per player. Therefore, 1) the mean of all four trials was recorded for analysis, and 2) the reliability of the time to complete the agility test could not be determined in this study. However, reliability (ICC and CV) of the time to complete the agility test was previously reported as 0.82 and 1.06% for a similar sample of soccer players [19]. Moreover, the CV of the tester’s movement time (time interval from the first movement of the ball to the moment the ball leaves the tester’s foot to the left or the right side) recorded by a video camera was 3.01% in the present study.

### Data analysis

The time required to complete the linear-sprint, change-of-direction, and agility test were automatically determined by the timing lights. The calculation of the perceptual-cognitive deficit was guided by the concept of the change-of-direction deficit, i.e., change-of-direction test time minus linear-sprint test time using similar distances, which has been recently introduced to performance testing [21]. Therefore, the perceptual-cognitive deficit was calculated as the difference between the time required to complete the change-of-direction and agility test as an indicator of the perceptual-cognitive processes that occur in response to the soccer-specific stimulus during the agility test.

### Statistical analysis

The data were analyzed using SPSS statistical software version 25.0 (SPSS, Inc., Chicago, IL). Mean values and standard deviations (SD) were calculated for each variable. Regarding the reliability of the tests (see *Procedures*), ICC values (absolute agreement, single measures) and CV values ((SD of all trials / mean of all trials) * 100) were computed.

Relationships between agility performance with linear-sprint (5 m, 10 m, and 30 m), change-of-direction performance, and the perceptual-cognitive deficit were determined by Pearson’s product-moment correlations (r) with 95% confidence intervals (95% CI), as normal distribution of the data was given. The magnitude of the correlation coefficient was considered as small (0.1 ≤ r < 0.3), moderate (0.3 ≤ r < 0.5), large (0.5 ≤ r < 0.7), very large (0.7 ≤ r < 0.9), and nearly perfect (r ≥ 0.9) [22]. The significance level for all statistical tests was set to 0.05.

## Results

Descriptive statistics (mean ± SD) of all tests are reported in Table 1. Linear-sprint performance and perceptual-cognitive deficit showed large correlations, and change-of-direction performance a very large correlation to agility performance, respectively (Table 2).

**Table 1:** Descriptive results for agility, 5-m sprint, 10-m sprint, 30-m sprint, change of direction performance, and perceptual-cognitive deficit.

	Agility	5-m sprint	10-m sprint	30-m sprint	Change of direction	Perceptual-cognitive deficit
Mean ± SD [s]	3.12 ± 0.08	1.01 ± 0.07	1.79 ± 0.08	4.40 ± 0.21	2.40 ± 0.06	0.73 ± 0.05

Results are presented as mean values and SD.

SD–Standard deviation

**Table 2:** Pearson’s r (r2), 95% CI and p-values for correlations between agility and 5-m sprint, 10-m sprint, 30-m sprint, change of direction performance, and perceptual-cognitive deficit.

	5-m sprint	10-m sprint	30-m sprint	Change of direction	Perceptual-cognitive deficit
Pearson’s r (r^2^)	0.57 (33%)	0.59 (35%)	0.69 (48%)	0.77 (59%)	0.55 (30%)
95% CI	0.20–0.82	0.17–0.87	0.28–0.89	0.43–0.96	0.13–0.87
p-value	0.03	0.02	< 0.01	< 0.01	0.03

r^2^ –Coefficient of determination; 95% CI– 95% Confidence interval

## Discussion

The purpose of this study was to extend our current understanding of factors underpinning agility performance in soccer by examining its relationships with not only physical (linear-sprint and change-of-direction performance) but also perceptual-cognitive factors (perceptual-cognitive deficit) in a newly developed soccer-specific agility test using a sample of amateur soccer players.

The main finding of this study was that physical factors showed large (linear-sprint performance, r = 0.57–0.69) to very large relationships (change-of-direction performance, r = 0.77), and perceptual-cognitive factors a large relationship (perceptual-cognitive deficit, r = 0.55) with agility performance. These results indicate the influence of both components on agility [6, 9]. Another finding of this study was that, regarding linear-sprint performance, relationships to agility were higher with increasing sprint distance. Based on the present results, our hypothesis that both physical and perceptual-cognitive factors are related to agility performance can be accepted.

In terms of the physical factors investigated, the present results are in line with previous research. In particular, a study in high-level youth soccer players showed that change-of-direction performance is more strongly related to agility (r = 0.58–0.64) than linear-sprint performance (r = 0.36–0.49) [17]. Similar results have been observed in high-level rugby league players (change-of-direction performance, r = 0.40–0.58; linear-sprint performance, r = 0.29–0.51) [12]. While all three types of tests examined comprise phases of linear sprinting, this finding seems plausible as agility tests include at least one change of direction, which indeed is true for change-of-direction tests but not for linear-sprint tests [21].

Despite the very large correlation between change-of-direction and agility performance in this study, there still remains a considerable unexplained variance (41%) between these two measures. According to our results, this variance can be partially attributed to perceptual-cognitive factors, indicated by a large correlation (r = 0.55; r^2^ = 30%) between the perceptual-cognitive deficit and agility performance. In particular, the perceptual-cognitive deficit introduced in this study reflects the sum of the response time (time interval from the live tester starting the timing to the player beginning to move forward) and the decision-making time (time interval from the moment the ball leaves the live tester’s foot to the left or the right side to the player initiating the directional change) during the agility test [19]. These measures have both been shown to contribute to agility performance in other sports such as Australian Rules football and basketball [23, 24]. Likewise, Altmann et al. [19] investigated the relationships between response time and decision-making time with total performance in the soccer-specific agility test which was also applied in the present study. Indeed, the authors reported only small to moderate relationships with agility when considering these two measures independently (response time, r = 0.25–0.32; decision-making time, r = 0.15–0.38). However, the higher relationship in the current study might be due to the perceptual-cognitive deficit reflecting a combination of both measures. Practically speaking, our results indicate that players with superior perceptual-cognitive skills would outperform other players with the same level of physical skills, however, with lower perceptual-cognitive skills in an agility task.

Nevertheless, the magnitude of correlations relating to physical factors in the present study was higher compared to findings commonly reported in the literature [12, 16, 17]. These differences could be attributed to the characteristics of the sample in the present study (adult amateur players) compared to those in the respective research literature (youth and adult high-level players). In addition, it is known relationships between test performances depend on the similarity of the tasks in question [25]. Consequently, further explanations for this result might be that the agility test in the present study 1) included only two directional changes at a relatively wide angle of about 45° and 2) used the same movement pattern as the change-of-direction test [14, 15]. Overall, these results re-emphasize the relatively high influence of physical factors on total performance in this particular agility test, as already indicated by Altmann et al. [19] (correlation of r = 0.70–0.72 between the movement time measured during the completion of the test and the total agility performance).

Another finding of this study is that longer linear-sprint distances yield higher relationships to agility compared to shorter distances (r = 0.57, 0.59, and 0.69 for 5-m, 10-m, and 30-m time, respectively). This finding might seem counterintuitive as the agility test involves short, explosive accelerations rather than maximum-speed phases. Yet, it is well in line with research by Krolo et al. [17] and Gabbett et al. [12], which showed a similar pattern for 5-m, 10-m, and 20-m linear-sprint times. A possible explanation in this regard might relate to lower body power. While lower body power is more strongly correlated to maximum-speed phases (i.e., 30 m) compared to acceleration phases (i.e., 5 m and 10 m) [26], it also contributes to effective changes of direction, which are required during the completion of an agility test [27]. However, as lower body power was not measured in the present study and as its influence depends on the characteristics of the specific task, this can only be speculated.

The main strengths of this study are the inclusion of an agility test tailored to the demands of soccer (i.e., high ecological validity) and that the change-of-direction test utilized the same movement pattern as the agility test. Based on recent research [6, 10, 28], this allows for more meaningful results compared to studies using unspecific stimuli [17] or investigating the relationships between agility and change-of-direction performance during tests that follow different movement patterns [12]. Moreover, using the same movement pattern during the agility and change-of-direction tests permits the calculation of the perceptual-cognitive deficit. As a measure of the players’ perceptual-cognitive processes that occur in response to the soccer-specific stimulus during the agility test, this parameter can be calculated without the need for a high-speed camera and the subsequent time-consuming data analysis. In doing so, the players’ agility performance can be distinguished into four classifications based on both their change-of-direction performance and their perceptual-cognitive deficit: fast mover/fast thinker, fast mover/slow thinker, slow mover/fast thinker, and slow mover/slow thinker [12].

Nevertheless, we did not investigate how using generic stimuli such as flashing lights would potentially affect our findings. Therefore, no conclusions can be drawn in this regard. It should also be noted that the present results were obtained in a relatively small sample of amateur soccer players using a novel soccer-specific agility test. Thus, the transferability of the findings to other populations and agility tests remains to be determined [27]. Moreover, as our investigation was cross-sectional in nature, no conclusions about causality can be drawn on this basis. In order to examine possible transfer effects, future studies should investigate the impact of physically-oriented (e.g., linear sprints, change-of-direction sprints) [29] as well as perceptual-cognitive oriented training interventions [30] on agility performance. Lastly, the players in the present study did not perform a habituation session. Although the players were used to tasks similar to our tests during training, and the tests showed acceptable intraday reliability in our study, a habituation session should be included in future studies.

## Conclusion

The findings of this study highlight the relatively high contribution of both physical (i.e., linear-sprint and change-of-direction performance) and perceptual-cognitive factors (i.e., perceptual-cognitive deficit) in relation to soccer-specific agility performance in amateur soccer players. Therefore, coaches and researchers are advised to include both elements in training programs aiming at improving agility performance at this playing level. Regarding physical factors, change-of-direction and maximum-speed drills should be prioritized because of their relatively higher influence on agility. Moreover, by applying a change-of-direction test that utilizes the same movement pattern as the agility test, the here introduced perceptual-cognitive deficit allows for a convenient and likewise thorough analysis (i.e., both physical and perceptual-cognitive factors) of agility performance, which is considered a key element for success in soccer.

## Supporting information

S1 Table Raw data. (XLSX)
